# Strategies and Techniques for Powering Wireless Sensor Nodes through Energy Harvesting and Wireless Power Transfer

**DOI:** 10.3390/s19122660

**Published:** 2019-06-12

**Authors:** Roberto La Rosa, Patrizia Livreri, Carlo Trigona, Loreto Di Donato, Gino Sorbello

**Affiliations:** 1 STMicroelectronics, Stradale Primosole 50, 95121 Catania, Italy; 2 Department of Engineering, University of Palermo, Viale delle Scienze Ed.9, 90128 Palermo, Italy; patrizia.livreri@unipa.it; 3 Department of Electrical, Electronics and Computer Engineering, University of Catania, viale A. Doria 6, 95126 Catania, Italy; carlo.trigona@dieei.unict.it (C.T.); loreto.didonato@dieei.unict.it (L.D.D.); gino.sorbello@unict.it (G.S.)

**Keywords:** WSNs, radio frequency, energy harvesting, wireless battery charger, lithium ion battery, wireless sensor networks, internet of things

## Abstract

The continuous development of internet of things (IoT) infrastructure and applications is paving the way for advanced and innovative ideas and solutions, some of which are pushing the limits of state-of-the-art technology. The increasing demand for Wireless Sensor Nodes (WSNs) able to collect and transmit data through wireless communication channels, while often positioned in locations that are difficult to access, is driving research into innovative solutions involving energy harvesting (EH) and wireless power transfer (WPT) to eventually allow battery-free sensor nodes. Due to the pervasiveness of radio frequency (RF) energy, RF EH and WPT are key technologies with the potential to power IoT devices and smart sensing architectures involving nodes that need to be wireless, maintenance free, and sufficiently low in cost to promote their use almost anywhere. This paper presents a state-of-the-art, ultra-low power 2.5 μW highly integrated mixed signal system on chip (SoC), for multi-source energy harvesting and wireless power transfer. It introduces a novel architecture that integrates an ultra-low power intelligent power management, an RF to DC converter with very low power sensitivity and high power conversion efficiency (PCE), an Amplitude-Shift-Keying/Frequency-Shift-Keying (ASK/FSK) receiver and digital circuitry to achieve the advantage to cope, in a versatile way and with minimal use of external components, with the wide variety of energy sources and use cases. Diverse methods for powering Wireless Sensor Nodes through energy harvesting and wireless power transfer are implemented providing related system architectures and experimental results.

## 1. Introduction

The markets for internet of things (IoT) devices and wireless sensor networks are highly promising, with a predicted trillion “things” to be connected by 2025 [1]. Smart systems and innovative solutions arouse also interest and typically require embedded sensing, actuating capabilities, signal processing, power generation and energy management. It is worth mentioning that the trend, more and more through the research and the adoption of novel materials, is to address flexibility, stretch ability, sustainable and environmental-free solutions. Certain persistent technical issues, however, need to be addressed before these technologies can truly be diffused as mature wireless solutions that are maintenance-free, sufficiently inexpensive and able to service vast amounts of sensor nodes [2,3,4,5]. Many wireless sensor network (WSN) platforms have been proposed to support infrastructures, home and office automation in order to enhance services, energy saving, comfort and security [6,7,8,9,10,11,12]. In the above scenario an extremely important issue is the requirement of reduced power consumption as well as the maximum area coverage [13,14]. To this purpose, license-free industrial scientific and medical (ISM) frequency bands have been adopted as the best solution to cover wide areas and at the same time cut down infrastructure costs: ZigBee [15], WLAN [16] and Bluetooth [17,18].

In order to cope with the cost requirements, and the wide variety of scenarios and use cases, we pursued an integrated ultra-low power system on chip (SoC) approach. Because the sources of energy to harvest are various and quite different with peculiarities that need to be specifically addressed, here we present a highly integrated, flexible and widely configurable SoC capable of harvesting multi-source energy and allow wireless power transfer (WPT).

An innovative platform based on a 2.5 μW ultra-low power SoC is presented. This high versatile solution is capable to perform multi-source energy harvesting (EH) through the integration of high performance ultra-low power circuitry. The SoC has been conceived as a novel modular system architecture and distinguishes itself as a very versatile platform for radio frequency (RF) energy harvesting and wireless power transfer that can be used to implement, with minimal effort, many practical use cases in real scenarios and different power sources. When integrated in a system, the SoC provides alternative methods for powering and easily maintaining WSNs. This is made possible with an innovative multipart system architecture that integrates a DC/DC converter with enable, a specific wide bandwidth RF to DC converter performing high power conversion efficiency (PCE) and one of the best in class low power sensitivity. Added value is given by the integration of an Amplitude-Shift-Keying/Frequency-Shift-Keying (ASK/FSK) receiver, an asynchronous finite state machine and a programmable logic circuit which renders the system configurable; being able to receive simultaneously data and power. Those features provide a high versatility to the system which can be configured, either on the fly or statically, to address different scenarios. All this makes possible to implement several different use cases where generally is needed to properly differentiate power strategies and behaviors.

The paper is organized as follows. Section 2 extensively discusses the previous works on WSNs, shows the issues faced so far by researchers and provides motivations for problems that still needs to be solved. In Section 3, details of the platform are explained. This section enlightens more details of the SoC, by showing its state-of-the-art performances, the specific architecture and key features. Section 4 shows how the original features of the SoC make easy to set up the different use cases reporting results of simulations and experiments. Specifically in Section 4.1, we describe how to nullify standby consumption with WPT. In particular, it is explained how the architecture of the SoC, with its unique key features and the advanced performances of its circuitry, achieves the wake up of a specific and completely off wireless node. It will be described how the high performances of the nano-power management and the low power sensitivity of the RF to DC converter allow a remote wake up of an off device from a distance of 8 meters and how the SoC architecture, that combines the programmable logic circuit, the ASK/FSK receiver and the DC/DC converter can select a specific node to wake up. In Section 4.2, Wireless battery charger based on WPT, it is discussed the use case of an over the distance wireless battery charger to show how the high PCE of the RF to DC converter can effectively achieve a convenient and efficient WPT. An interesting feature is also explained, which shows how the SoC can be remotely controlled to easily switch between different use cases with no rework of the board and no extra external components. Finally, in Section 4.3, powering battery-free devices with WPT, the paper shows how to use the SoC in set-and-forget battery-free systems where different sources of energy, such as RF or photovoltaic, are available. It is also proved how the high performance of the nano-power internal circuitry offers the advantage, compared to existing solutions, to use small and low cost devices, how the integration of the RF to DC converter avoids the use of external diodes, leading to a compact solution, and how the SoC permits multi-source for both EH and WPT. It is also highlighted how due to the integration in the SoC of the RF to DC converter, the ASK/FSK demodulator and the asynchronous finite state machine, the system is able to establish a closed loop communication with the IoT node [19] gaining the advantage to get an optimized minimum duty-cycle. Conclusions follow.

## 2. Related Work

The wireless node has evolved from a simple tag transponder used principally for item identification to an advanced device also able to perform sensing and computation. For advanced WSNs, the ability to pre-process data where and when it is collected is an increasingly critical feature [20]. The evolved node, of course, requires more energy, which in many cases is provided by a battery that eventually needs to be replaced, incurring additional costs and interrupting operation. To save energy, low-power and low-cost technologies such as bluetooth low energy (BLE) [21] are gaining momentum in industrial WSNs, in alternative to the industrial profiles for the IEEE 802.15.4 standard [22,23], and considerable research has recently focused on how to support real-time communications over star [24] and multi-hop mesh [25] BLE networks for industrial IoT. Nevertheless, battery life poses a real problem in node maintenance as the task is often costly and difficult to perform, and at times even impossible because sensor nodes are often positioned in inaccessible places. To help render IoT truly ubiquitous, sensor technology is required that enables the deployment of a large number of wireless, easy maintenance and inexpensive sensing devices that can be used almost anywhere [26,27]. All this points to the use of regenerative EH and WPT where possible [28,29]. In order to address certain maintenance issues in IoT and WSN, different strategies depending on the kind of system are possible: some systems, such as those implementing real time sensors, may require batteries; other systems, such as low duty cycle sensors [30] and measurement architectures [31], may be able to function appropriately without batteries. In a recent example, nodes that had to be embedded in concrete structures, adopted, with energy-efficient solutions, a battery-less, wireless-power sensor to ensure a maintenance-free life cycle [32]. In many other applications, the necessary power for sensing or computation can only be provided by a local battery. In applications involving dedicated short range communication (DSRC) used, for example, in toll devices, a battery powers the integrated circuits, while the actual communication takes advantage of the modulation of power back-scattered by the antenna [33]. In battery-powered devices, the use of dedicated, circularly-polarized antennas [34,35] can only improve polarization match and directivity, which will increase communication range but will not reduce power consumption. A significant reduction in battery power consumption can, on the other hand, be achieved by reducing or eliminating standby power consumption [36,37,38,39,40]. Power management circuits are in fact permanently on standby, and depending on the duty cycle, can consume unnecessarily high percentages of battery charge [41]. The null standby leads to a significant saving which can be translated into prolonged battery life or in a reduction of battery size, which is beneficial in terms of cost and system miniaturization [37]. Even after nullifying standby power consumption, however, batteries will eventually be drained of all their charge. In WSNs, node maintenance due to battery replacement is often highly undesirable as nodes can be difficult or impossible to reach. For this reason, the most convenient way to facilitate the maintenance of battery-powered nodes is through the implementation of an over-the-distance wireless battery charger using RF WPT [42].

Where applicable, such as in low duty cycle sensors, the ultimate solution to node maintenance is to eliminate it entirely through set-and-forget, battery-free sensors [32,43], which wipes out the limitation of system lifetime caused by batteries, and render devices non-disposable, more cost-efficient and functional as long as power is available or delivered [44].

Recent advances in ultra-low power chip design techniques have enabled a deep research toward long lifetime WSN devices performing complex applications with SoCs that achieve good integration and even energy harvesting. For example, Ref. [45] presents an SoC to harvest 19 μW for battery-less energy harvesting body sensor nodes (BSN), the system in [46] integrates several chips with solar cells and a battery to accomplish near-perpetual operation for measuring intraocular pressure. Ref. [47] presents a multi-source 6.45 μW self-powered SoC for solar and thermoelectric generator (TEG) energy harvesting and Ref. [48] shows a developed chip to scavenge energy from artificial light.

A solution to all of the above issues through a versatile SoC is addressed in the following sections. Solutions are discussed both in terms of functionality and performance to implement EH and RF WPT, for use as either an RF power receiver or for ultra-low power, high performance, power management for multi-source EH.

## 3. System Description

The SoC platform is based on a device that integrates an RF high performance, wide-bandwidth, RF to DC energy transducer (350 MHz–2.4 GHz), with −18.8 dBm sensitivity at 900 MHz, a maximum power efficiency of 45% at 900 MHz with an input power of −10 dBm, and an accurate ASK/FSK demodulator with a modulation index as low as 10% and a sensitivity of −38 dBm [49,50,51].

**Figure 1:**
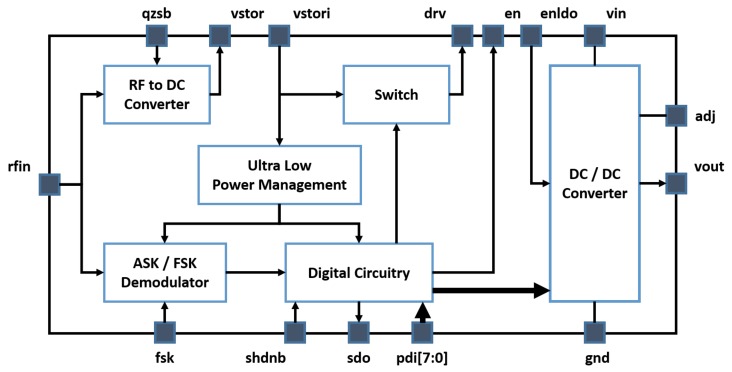
System on chip (SoC) block diagram.

With reference to Figure 1, the ultra-low power management is the key circuitry to scavenge the energy with a nano-power circuit whose quiescent current is as low as 75 nA. It is an intelligent power management as it is able to both measure the scavenged energy and provide a regulated voltage to bias the internal micro-power circuitry of the SoC as soon as the harvested energy is enough to guarantee the energy requirement of the active phase of the system. In order to address and properly configure the IoT device the SOC is equipped with an ASK receiver that supports both the 433 MHz and 915 MHz ISM bands. In order to comply with European regulation ETSI 300-200.1 it has been designed for a minimum data rate of 62 kbit/s to allow a channel bandwidth occupation of around 250 kHz. Since Manchester decoding can be performed with simple, low power, digital circuitry based on time-delay cells, this coding has been adopted to both reduce channel bandwidth occupation and system complexity. The system integrates an ultra-low power digital circuitry to allow several other features and a digital programmable DC/DC converter to provide, if needed, a regulated voltage supply to an IoT node.

It is worth highlighting that for long distance WPT, the 900 MHz range could be the best trade-off. The main reason is that European regulation ETSI 300-200.1 allows transmitting up to 27 dBm providing a good start in terms of power availability. Moreover, this frequency is fairly good in terms of antenna size which is important for IoT devices which often show miniaturization issues. A lower frequency such as 433 MHz is certainly advantageous for a lower free space path loss (FSPL) but the power allowed in transmission is also much lower, and this choice would provide miniaturization issues for devices like WSNs. Higher frequencies such as 2.4 GHz provide a higher FSPL and the allowed power that can be transmitted is limited to +20 dBm, however this frequency certainly offers an advantage in terms of system miniaturization since the antenna design can be more efficient. For this reason, it not always easy to understand which is the best option, and the choice often arises from a trade-off between allowed power that can be transmitted, FSPL and the specified size of the system.

## 4. Results

### 4.1. Nullifying Standby Consumption with WPT

Wireless data, in the form of wireless sensor nodes and IoT devices, demands wireless power. To extend energy autonomy in IoT devices it is necessary to reduce energy consumption as much as possible. A big impact in the energy consumption of a wireless node or remotely controllable appliances is given by the standby power consumption. In fact, even if the power consumption of the electrical appliances in standby can be quite low, in the majority of the use cases power management circuits continue consuming energy unnecessarily for a long time, resulting in a not negligible energy consumption. To avoid this, an approach is proposed where appliances are effectively turned off, never stay in standby, and finally are remotely woken up by RF WPT. The basic premise is that with careful design a very small amount of energy is needed to wake up a completely off device. Thus, considering the high distance attenuation and limited transmission power of the remote unit, the received RF energy is never very high but can still be converted into sufficient electrical energy to switch on an electrical appliance by doing a proper design of the ultra-low power receiver in terms of power sensitivity and efficiency. The proposed solution for sensor nodes based on SoC includes an RF to DC energy transducer with a power sensitivity of −18.8 dBm. This level of power sensitivity is one of the best performances achieved in SoC and allows us to greatly extend the maximum allowable distance between nodes up to the convenient target of eight meters when operating at 900 MHz with a transmitted power of 0.5 W in free space. The system requires ultra-low power management with a quiescent current below 100 nA to allow self-powering operation with a current as low as 1 μW in the worst case sensitivity condition. The SoC exhibits the unique and novel feature that allows to specifically address the remote node to be woken up. For this purpose it integrates an ASK/FSK receiver and a programmable digital circuitry to recognize the address of the node to be powered. Another element of novelty is the integration of an asynchronous finite state machine that allows to keep the wireless node powered with the battery, as long as the node needs to stay on, even when the RF powered is faded away.

**Figure 2:**
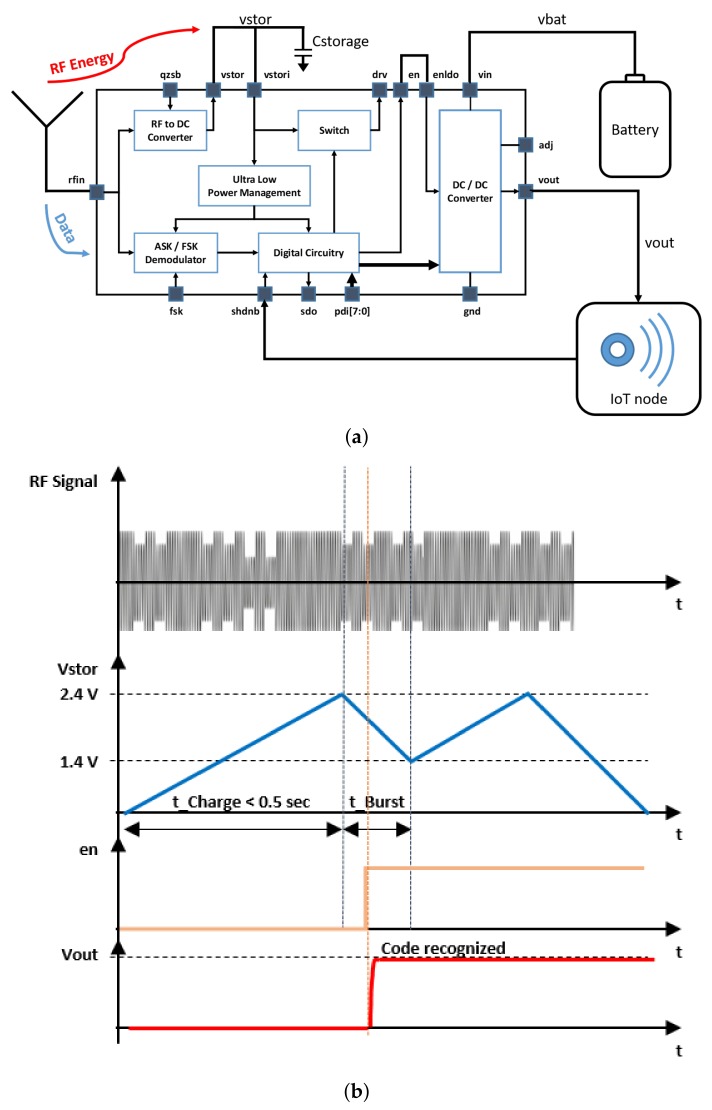
Remotely controlled internet of things (IoT) device with no standby consumption: (**a**) block diagram, (**b**) timing diagram.

Figure 2a, shows how a battery supplies a real time IoT device through a remotely controllable DC/DC converter integrated in the SoC. As shown in Figure 2b, when the system is off, no battery power is consumed being all the other units in the IoT device completely off as the ($V_{\mathrm{out}}$) regulated voltage of the DC/DC converter is zero. The system is woken up by transmitting an RF signal through a remote control unit. The transmitted power must be enough to turn the system on within a specified time according to the target distance and the receiver sensitivity. (0.5 W from a distance of eight meters at 900 MHz in free space for no more than 500 ms with a receiver sensitivity of −18 dBm). The energy caught by the RF antenna is converted by the RF to DC converter and stored in the external capacitor $C_{\mathrm{storage}}$, the voltage ($V_{\mathrm{stor}}$) across it biases the internal circuitry of the SoC. Once the bias voltage is above a defined programmable threshold which defines that the stored energy is enough for the internal circuitry of the SoC to operate, the demodulator and the digital decoder are enabled to check if the address of the device is correct and only in this case the DC/DC converter is turned on (the EN signal goes high) providing the voltage ($V_{\mathrm{out}}$) to rise and bias the rest of the system (IoT node). Since now on, the battery begins to supply power, after enabling the system to let it work even when the power transmitter is no longer emitting the “en” signal, is latched to stay high as long as the equipment needs to operate. The “shdnb” pin can be used to reset the logic section once the equipment has finished its job and the system can be turned back off. In this case, the “en” signal goes low and the power path between the battery and the output of the DC/DC converter is in high impedance, implying a negligible leakage current consumption of few pA to ensure zero-energy “stand-by” operation or a fully turned-off device [37].

It is worth noting that to increase the maximum distance from which an off device can be woken up, for given transmitted frequency and antenna gain, the main parameter to optimize is the power sensitivity of the RF to DC converter. Very important is also the way the power is transmitted while performing WPT as channel fading and proper signal design enhances the RF to DC conversion efficiency of non-linear RF energy harvesters [52,53].

### 4.2. Wireless Battery Charger Based on WPT

As shown in the previous section the aim to extend the lifetime of battery-supplied nodes can be pursued by minimizing the power consumption of electronic. This can certainly be achieved through an accurate design and research of low or ultra-low power components and by nullifying standby power consumption where possible. Unfortunately, in battery driven devices, maintenance will at some point still be needed since for these kinds of devices, by nullifying standby power consumption, the maintenance operation can only be postponed as much as possible. One of the proposed ideas is to use WPT with RF energy to charge the batteries over the distance with the intent to prevent or greatly reduce the cost of labor of battery replacement. By nullifying standby power consumption maintenance schedules can be reduced while charging batteries over the distance would lower battery replacement costs. While RF energy offers the advantage of being highly pervasive with the possibility to be conveniently transferred to out-of-sight sensing devices, the efficiency of the power transfer is actually quite low, this explains why this technique is limited to ultra-low energy devices. As well shown by the equation for Friis transmission [54], for a typical power transmission in the range of 900 MHz, in free space, the transmitted power decays of −30 dB (1/1000) after a distance of only one meter and continues decaying −20 dB every 10 meters. Even if this suggests that RF WPT is really far from being efficient, RF energy transfer establish itself as a very convenient way to provide power to small energy devices such as IoT and wireless sensor nodes, where power consumption and dissipated energy levels represent important factors that need to be addressed [13]. The amount of RF energy that can be stored in the battery in a defined amount of time depends on many parameters, including transmitted power, the gain of the receiving and transmitting antenna, the frequency of the transmitted RF signal, the distance between the RF transmitter and the efficiency of the RF Harvester. In free space, the RF power available at the antenna terminals can be calculated with the following equation for Friis transmission:

(1) $$P_{A}=P_{T}\frac{G_{T}G_{R}\lambda^{2}}{{\left(4\pi d\right)}^{2}}\tau ,$$

where $P_{A}$ is the available power, $P_{T}$ is the transmitted power, $G_{T}$ the transmitting antenna gain, $G_{R}$ the receiver antenna gain, λ the wavelength, τ is the polarization efficiency [55] (2.282) and *d* the distance between receiver and transmitter antenna. In (1), we can simply assume an ideal polarization match and τ=1. This is approximately true in many applications, where circularly polarized antennas are used [34,35] otherwise an appropriate (less than 1:1) polarization efficiency should be considered. The receiving antenna efficiency, $\eta_{R}$, is taken in account by $G_{R}=\eta_{R}D_{R}$, where $D_{R}$ is the antenna directivity [55] (2.107). In general, not all the available power is delivered to the rectifier as some power is reflected back and the received power is $P_{R}={\left(1-|\Gamma \right|}^{2})P_{A}$. If we assume a perfect conjugate match and no losses other than the antenna losses already considered, we can assume Γ≈0 and $P_{R}=P_{A}$. Finally, the power delivered to the battery is given by the product of the received power ($P_{R}$) at the antenna and the efficiency of the RF harvester ($\eta_{H}$) as follows:

(2) $$P_{\mathrm{bat}}=\eta_{H}P_{R}.$$

**Figure 3:**
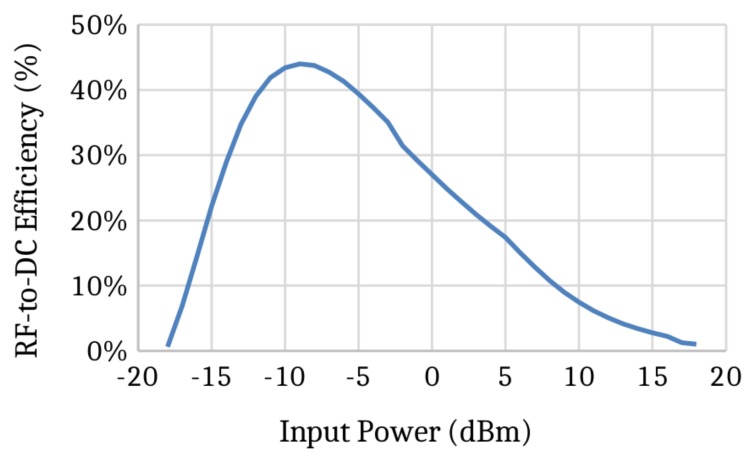
Radio frequency (RF) to DC efficiency vs. input power [42].

Thus, considering a distance of two meters between the transmitter and receiver in free space conditions, a transmitted power of 0.5 W (27 dBm) at a frequency of 900 MHz and gain of 1 for both antennas ($G_{T}=G_{R}=1$), the received power equals 88μW (≈−10.5 dBm). Since, as shown in Figure 3, the efficiency of the RF to DC transducer is approximately 40% for $P_{R}\;=\;-10.5$ dBm, the power delivered to the battery ($P_{\mathrm{bat}}$) is 35 μW with 20 μA average current delivered to a 2.3 V battery.

**Figure 4:**
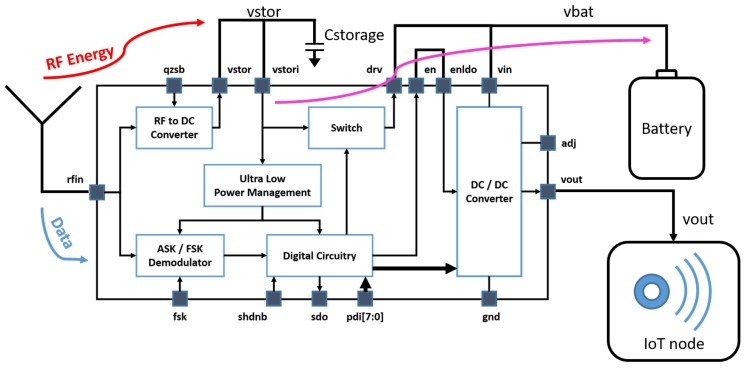
SoC as wireless battery charger.

In WPT, the antenna gain is crucial, and from (1) we can see that it directly impacts the received power and consequently the sensing architecture or measurement system performance. For this purpose, a dedicated high gain antenna architecture is proposed, as in [56]. In the block diagram in Figure 4, the RF energy charges the battery directly through the switch integrated in the SoC.

**Figure 5:**
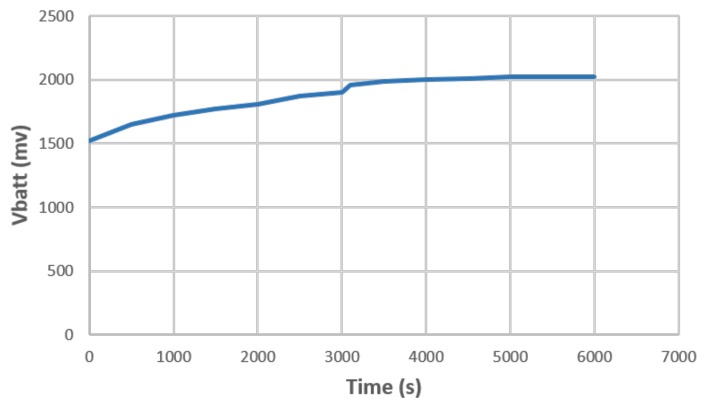
Battery voltage vs. time with power transmitter and receiver at distance d=170 cm.

Some experimental results are given in Figure 5, where we show the charging curve for the commercially available 2.3 V and 3 mAh UMAC battery provided by MuRata [57], that was charged via the STMicroelectronics SPIRIT1 [58] sub-GHz transceiver performing power transmission placed 170 cm away from the SoC developed for power reception. The power transmitter was programmed to deliver 27 dBm of power at 900 MHz, while the voltage and current delivered to the battery were constantly monitored every five seconds on the power receiver side. Both the power transmitter and the RF energy harvester are equipped with the Revie Pro antenna provided by Laird [59]. At the distance of 170 cm, the average measured current was about 20 μA and it took 30 min to charge the battery from 1.6 V to 2.1 V.

It is worth pointing out that for this use case, for given transmitted frequency and antenna gain, it was fundamental to optimize the PCE of the RF to DC converter. Techniques referred to as maximum power point tracking (MPPT) can be used to determine the maximum power available from these systems. Since those techniques are very well-known and used in low power systems, in ultra-low power systems MPPT are normally neglected for the absence of a convenient way, from the energy investment point of view, and the general opinion is that since the power involved is so tiny, the extra circuitry involved to optimize the power conversion could have a power consumption not negligible and eventually would not make it worthwhile [60,61].

Even in this case the SoC shows the unique and original feature that the power flow can be diverted from the $C_{\mathrm{storage}}$ capacitor into the battery through the programmable internal switch. This is quite novel and it is interesting to see how, by sapient use of WPT, the configuration can be done on the fly, by simply using the data sent through the modulated power carrier. It should be noted that the system can be used in its normally way being woken up from the off state, as seen in the previous section, and switched to the wireless battery charger state, as soon the battery charge is low, by simply sending different commands through the data of the modulated power carrier. This powerful feature allows high versatility and remote operation with no rework of the board and no extra external components to switch from one use case to the other.

### 4.3. Powering Battery-Free Devices with WPT

A battery powered sensor node is a disposable item whose use is strictly limited by the life span of the battery. The main consequence of this is the high maintenance cost of the wireless sensor networks, especially when, as it is often the case, the sensors are placed out of reach [32] or in hazardous places. The preferred solution, when and where applicable, is to use sensors that do not require batteries so that their lifetime is not limited because of the battery. This solution requires power architecture that is specifically designed for wireless sensor nodes, with ultra-low power management able to handle currents in the order of nano-amperes.

The energy may derive from several sources, including RF, thermal, photovoltaic, vibrational, etc. Among these, the most interesting is RF WPT due to its availability and the pervasiveness of RF energy, which can penetrate zones that are out-of-sight. Also in this case, it should be remembered that the efficiency of the power transfer is very poor, and that this technology is generally useful for low energy devices. Moreover, because of the high attenuation of the transmitted power with the frequency and the distance between transmitter and receiver, the technology is primarily for applications where the distance is inside a few meters, at a frequency of 900 MHz.

**Figure 6:**
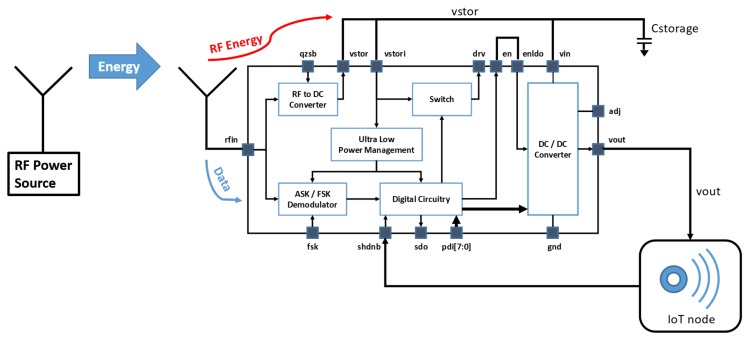
SoC as power source in battery-free use case.

**Figure 7:**
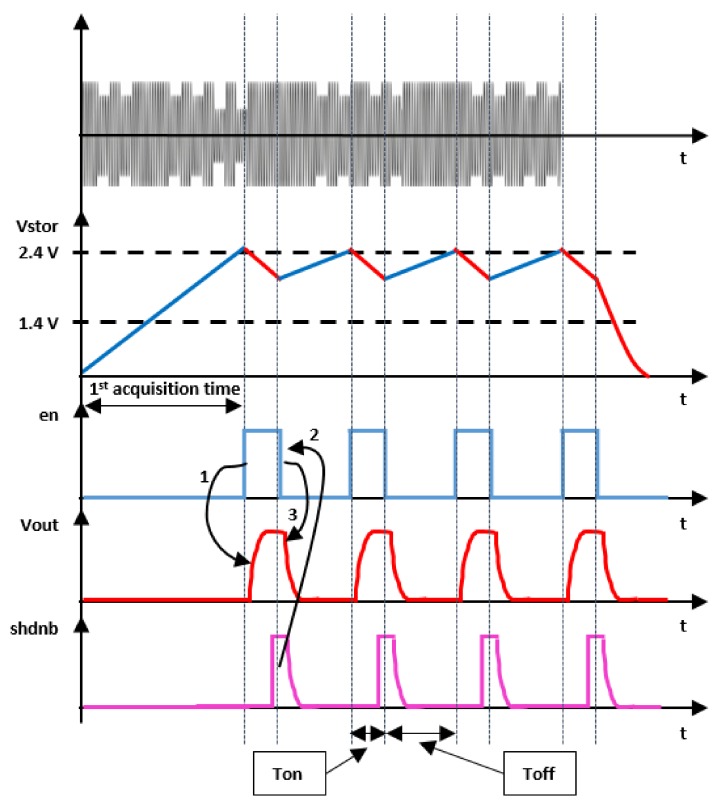
SoC power sequence in battery-free use case.

With reference to Figure 6 and Figure 7, the incoming radio frequency energy is converted by the RF to DC energy transducer into a current that charges the capacitor $C_{\mathrm{storage}}$ and causes a build-up of voltage $V_{\mathrm{stor}}$. When the voltage $V_{\mathrm{stor}}$ reaches the maximum voltage of 2.4 V, all the other internal SoC circuits turn on and the “enldo” signal goes high, which switches on the DC/DC converter that provides the regulated voltage $V_{\mathrm{out}}$ to the other units of the sensor node.

At this point, the system is completely on with a higher consumption that causes the voltage $V_{\mathrm{stor}}$ to drop because of the imbalance between the harvested and required energy. In fact, the $C_{\mathrm{storage}}$ capacitor is usually required to supply a current to the load in the order of milli-amperes, which is much higher than the harvested current that is typically in the order of micro-amperes. As soon as the IoT device finishes its activity, it rises up the signal “shdnb”, which triggers the internal finite state machine of the SoC to reset the “en” signal, causing the DC/DC converter to turn off and the voltage $V_{\mathrm{out}}$ to fall down. Since the voltage $V_{\mathrm{out}}$ is down and the IoT node is no longer biased the signal “shdnb” goes down as well. The advantage is that the drop of the energy stored in the $C_{\mathrm{storage}}$ capacitor is minimized according to the energy requirement of the IoT node, which could be case by case different. Since the drop of the voltage $V_{\mathrm{stor}}$ is minimized, the system is ready earlier to start scavenging energy and as a consequence the duty-cycle gets minimized. This is an original feature provided by the SoC which is able to establish a closed loop communication with the IoT node [19]. Due to this, the wireless node can alert the SoC when it has finished its activity and no longer needs energy so that the system can quickly switch back to the energy scavenging phase with the result to have an optimized management of the energy that eventually results in a reduced duty-cycle.

As discussed previously for the wireless battery charger based on WPT, at a distance of two meters with a transmitted power of 0.5 W at 900 MHz and a gain of one for both receiving and transmitting antenna, the average current charging the storage capacitor $C_{\mathrm{storage}}$ is about 20 μA. Considering an IoT device with a 1.8 V bias voltage that needs an average current of 10 mA in a time window of 50 ms would lead to choose a maximum voltage drop across the storage capacitor of 0.4 V which guarantees that the bias voltage of the IoT device never drops below 2.0 V which is important to provide margins to the voltage headroom for the low power electronic devices that typically require a bias voltage of 1.8 V. The $C_{\mathrm{storage}}$ capacitor needed to store the energy can be a small 1 mF capacitor with the only technology requirement to be very low leakage. The presented SoC configured as a battery-free sensor node, with a distance of two meters between the power transmitter and receiver, in free space transmission, with transmitted power of 27 dBm at 900 MHz would get a charging current of about 20 μA at the $C_{\mathrm{storage}}$ capacitor. Being the capacitor completely discharged, the sensor unit will be able to perform its first acquisition 120 s after the beginning of the power transmission. All acquisitions after the first, as long as the RF power transmitter remains on, are performed in much less time, being the $C_{\mathrm{storage}}$ capacitor pre-charged. With a maximum allowed voltage drop of 0.4 V, the maximum time needed to obtain all subsequent acquisitions is 20 s in the worst case condition.

**Figure 8:**
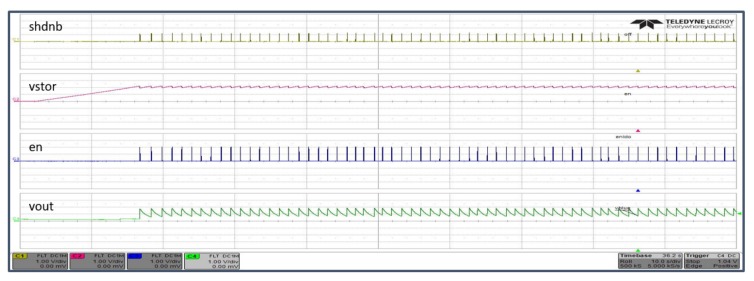
SoC power sequence in battery-free use case with power source 120 cm away.

Figure 8 shows experimental results in which the power transmitter was programmed to deliver 27 dBm (0.5 W) at 900 MHz. The wave-forms show the SoC power sequence, the voltage delivered to bias the IoT node, the first acquisition time and the duty-cycle with the power transmitter 120 cm away.

### 4.4. Powering Battery-Free Devices with PV Cell

**Figure 9:**
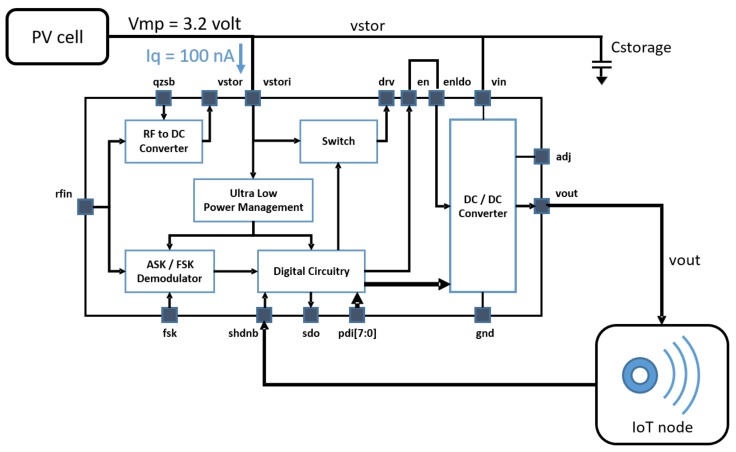
SoC as photovoltaic (PV) energy harvester in battery-free devices.

The SoC can be used for EH from several different sources of ambient energy, including vibrational, thermoelectric, photovoltaic, etc. Figure 9 shows the specific example of EH with a photovoltaic cell.

The ultra-low power management circuitry of the SoC intelligently manages the harvested energy provided by the photovoltaic cell. It delivers a regulated power supply to the internal circuitry of the SoC as soon as the amount of energy can ensure active operation of the system. The power management consists of nano-power circuitry that performs voltage sensing on the storage capacitor to control the charging process and micro-power circuitry that provides the regulated power supply to all the other internal parts of the SoC. During the charging phase, when the voltage $V_{\mathrm{stor}}$ across the $C_{\mathrm{storage}}$ capacitor is below the programmable threshold $V_{\max}$ (2.4 V or 3.2 V), the current consumption is as low as 75 nA. Once the voltage $V_{\mathrm{stor}}$ reaches its maximum $V_{\max}$, the current consumption of the power management unit increases to micro-amperes and the voltage across the capacitor $C_{\mathrm{storage}}$ drops, in the worst case, down to a $V_{\min}$ programmable threshold. As soon as the $V_{\mathrm{stor}}$ voltage reaches the threshold $V_{\min}$, the micro-power circuitry is turned off, which enables a new charging phase. With such low current consumption (75 nA) in the charging phase, a photovoltaic cell with a current capability of just 1 μA can easily charge the storage capacitor $C_{\mathrm{storage}}$. This allows EH with small, inexpensive photovoltaic cells, which is essential for the miniaturization of IoT devices in environments that are low in ambient light.

## 5. Conclusions

Different strategies and solutions for powering wireless sensor nodes across diverse applications and scenarios were analyzed for their benefits and limitations, with particular focus on RF EH and WPT. It was highlighted that even if RF EH still needs to evolve to become effective, RF WPT on the other hand allows battery-free, low duty-cycle sensors, and represents an efficient strategy to increase the battery life of remotely controlled devices and conveniently recharge batteries over distances for real time, battery driven devices. It was also demonstrated that ambient EH in combination with efficient power management SoC solutions allows no maintenance, low cost, battery-free smart sensors. Generally speaking, the techniques presented here can be viable as both individual solutions and in combination to significantly drive the development of very low maintenance and set-and-forget WSN.

A specifically designed SoC was also presented, with the proper functionalities and performances to allow diverse solutions for easy or no maintenance nodes in a wireless sensor network. System design considerations and application examples in several configurations ranging from RF and non-RF EH to WPT for both battery and autonomous or quasi-autonomous nodes were provided. The study represents a contribution to the effort towards the energy sustainability in the IoT and in WSNs. The experiments provide empirical evidence that WPT and EH techniques can be used to extend battery life, recharge batteries and ultimately allow battery-free devices. In this context it should be noted that in the future the research will deal with solutions which will complement silicon-based devices. In particular, new materials are needed for guaranteeing a significant diversification and the design of novel intriguing and smart solutions. It should be noted that in the perspective to realize power transfer architectures for autonomous or quasi autonomous systems ecological friendly and green materials, such as novel families of polymers and compounds will play a relevant role in the development of smart systems. In this context the trend that regards the research and development of new materials [62,63] is interesting and necessary in order to obtain disposable devices that are greener, low cost and recyclable.
